# Frequency and Association of Hyperuricemia in Non-alcoholic fatty liver disease patients: Still a neglected combination?

**DOI:** 10.12669/pjms.41.4.10333

**Published:** 2025-04

**Authors:** Ali Haroon, Saima Askari, Anita Haroon, Zahabia Sohail

**Affiliations:** 1 Ali Haroon, MRCP, FCPS, FRCP, Senior Registrar, Gastroenterology, Hamdard University Hospital and Consultant, Aga Khan University Hospital, Karachi, Pakistan; 2 Saima Askari, MBBS, FCPS, FCPS, Consultant Endocrinologist & Assistant Professor, Baqai Institute of Diabetes & Endocrinology (BIDE), Baqai Medical University, Karachi, Pakistan; 3 Anita Haroon, MRCP, FCPS, FRCP, Assistant Professor Department of Nephrology, Baqai Institute of Diabetes & Endocrinology (BIDE), Baqai Medical University, Karachi, Pakistan; 4 Zahabia Sohail, MBBS Post Graduate Resident, Department of Gastroenterology, Aga Khan University Hospital, Karachi, Pakistan

**Keywords:** Hyperuricemia, Non-alcoholic fatty liver disease, NAFLD severity

## Abstract

**Objective:**

To determine the frequency and association of hyperuricemia in non-alcoholic fatty liver disease patients.

**Method:**

This retrospective study was conducted at multiple healthcare facilities including Baqai Institute of Diabetology and Endocrinology (BIDE), Fatima Hospital and Imam Clinic over a span of one year (July 2023 to June 2024).This multi-center approach allowed for a comprehensive analysis and ensured diverse participant representation. The study focused on patients over 18 years old with known cases of non-alcoholic fatty liver disease (NAFLD) visiting outpatient departments. Demographic details, co-morbidities, examination findings, and pertinent laboratory tests were systematically documented utilizing a standardized proforma. Additionally, ultrasound reports of liver scans were scrutinized to categorize NAFLD severity into mild, moderate, and severe cases.

**Results:**

In this study of 246 NAFLD patients, severity distribution was 35% mild, 24.4% moderate, and 40.2% severe, with a mean age of 53.1 years and a female majority (52%). Significant associations were found between NAFLD severity and age, gender, blood pressure, tobacco use, diet, and obesity. Biomarker analysis revealed elevated levels in severe NAFLD cases, particularly uric acid (7.63 vs. 6.6 vs. 5.96; P<0.001) and HbA1c (6.77 vs. 6.15 vs. 5.8; P<0.001). Hyperuricemia was significantly associated with NAFLD severity (P: 0.001), with 56.6% of severe cases exhibiting hyperuricemia. Univariate logistic regression identified hypertension, diabetes, obesity, and severe NAFLD as significant factors for hyperuricemia (P: 0.0001).

**Conclusion:**

A significant prevalence of hyperuricemia was noted among NAFLD patients, underscoring the importance of integrating uric acid assessments and appropriate management into NAFLD care protocols.

## INTRODUCTION

Metabolic syndrome is associated with a clinical spectrum of liver abnormalities collectively known as nonalcoholic fatty liver disease (NAFLD).^1^ NAFLD is now considered a prevalent form of chronic liver disease, characterized by fat accumulation inside hepatocytes without significant or absent alcohol intake. Being a multi-factorial disease NAFLD is occurred by the interactions of genetics, diet, and lifestyle.^2^ Its clinical manifestation varies from hepatic steatosis to inflammatory steatohepatitis leading to fibrosis, cirrhosis, and or hepatocellular carcinoma. In developed countries, it affects greater than twenty-five % of the population.^3^ The local studies done in Lahore and Khyber Pakhtunkhwa show the prevalence of NAFLD at 41.37%^4^ and 47%^5^ respectively. Whereas another community-based study revealed a 60.8% prevalence in the urban population of Karachi.^6^

A growing body of observational data points to a significant association between serum uric acid (SUA) levels and the risk of NAFLD.^7^,^8^ The main end product of purine metabolism is uric acid, and an imbalance between uric acid production and excretion can lead to excessive levels of SUA in the body.^9^ According to several human investigations, NAFLD patients have high serum levels of the enzyme xanthine oxido reductase, which catalyzes the production of uric acid, and this enhanced production of uric acid can speed up the progression of NAFLD. By encouraging the overexpression of pro-lipogenic enzymes and proteins that bind to sterol regulatory elements, elevated uric acid levels may cause triglyceride buildup.^10^

Hyperuricemia is associated with a higher degree of liver damage along with proven cardiovascular risk factor. To the best of our knowledge, local data regarding the frequency and association of hyperuricemia in NAFLD patients is scarce. By knowing the frequency and association of hyperuricemia in NAFLD patients will increase the understandability of healthcare professionals for early screening of uric acid levels and potential uric acid-lowering therapeutic strategies to improve the outcome of the disease. Therefore, the objective of this study was to determine the frequency and association of hyperuricemia in non-alcoholic fatty liver disease patients.

## METHODS

This retrospective multi-centred study was conducted at Baqai Institute of Diabetology and Endocrinology (BIDE), Fatima Hospital Baqai medical university (BMU), and Imam Clinic Karachi during July 2023 till June 2024.

Data of all patients of both genders, aged >18 years, known cases of NAFLD visiting the outpatient department were enrolled in this study. However, patients having renal disease, pregnancy, malignancy, co-existing causes for chronic liver disease (viral hepatitis, autoimmune hepatitis, alcoholic liver disease, Wilsons, Hemochromatosis, Inborn errors of metabolism,) on drugs causing liver steatosis (mipomersen, lomitapide, amiodarone, methotrexate, tamoxifen, corticosteroids, valproate, antiretroviral medicines) using drugs causing hyperuricemia (thiazide diuretics, loop diuretics, cyclosporine, tacrolimus antituberculosis drugs) and/or incomplete records were excluded.

### Ethical Approval:

After taking the institutional review board (IRB) approval dated July 5, 2023 (Reference No.: 007/05/23/005), the data was collected from hospital record files of respective hospitals.

Demographic details, known co-morbidities, history of significant illnesses, and smoking status were noted. Blood pressure, height, and weight were recorded and body mass index was calculated by the weight in kg, divided by the square of their height in meters. The laboratory tests were included HbA1c, Liver function test (Total Bilirubin, Direct Bilirubin, Alanine aminotransferase (ALT), Aspartate aminotransferase (AST), Gama glutamyl transferase (GGT), Alkaline phosphatase (Alk Phos), Serum uric acid, fasting lipid profile, Serum creatinine, and the reports of ultrasound abdomen were noted. Ultrasound B-mode imaging allows to subjectively estimate the degree of fatty infiltration in the liver. All the reports were verified to be provided with imaging attached, reported by a qualified sonologist. All these details were recorded by using a pre-designed proforma.

Values considered Normal of all the parameters were as follow: HbA1c < 5.7 %; total bilirubin <1.2 mg/dl; ALT < 45 IU; AST < 35 IU; GGT< 55 IU, ALK < 130 IU; serum creatinine < 1.0 mg/dl, serum cholesterol <200mg/dl, triglyceride < 150mg/dl, HDL > 40mg/dl, LDL < 100mg/dl, VLDL < 30 mg/dl.

### Operational definition:

NAFLD is defined as macro vesicular steatosis ≥ 5% of hepatocytes in the absence of a readily identified alternative cause of steatosis (eg, medications, starvation, monogenic disorders) in individuals who drink little or no alcohol.^11^

### Severity of NAFLD:

The grading of liver steatosis is obtained using ultrasound (US) features that include liver brightness, contrast between the liver and the kidney, US appearance of the intrahepatic vessels, liver parenchyma and diaphragm. Mild NAFLD, if there is a mild and diffuse hyper echogenicity of liver and normally visualized diaphragm and the portal vein wall.^12^ Moderate NAFLD, if there is moderate increase of liver echogenicity and slightly decreased visualization of diaphragm and the portal vein wall.^12^ Severe NAFLD, if there is marked hyper echogenicity of liver and poor or no visualization of portal vein wall, diaphragm, and posterior part of the right liver lobe.^12^

### Obesity:

Obesity is defined as BMI > 25Kg/m^2^ as per World Health Organization cutoffs for Asian population.^13^ Hyperuricemia (HUA), Serum urate concentrations exceeding 7 mg/dl in males and >6 mg/dl in females is considered as hyperuricemia.^14^

### Statistical analysis:

Statistical analyses were conducted on statistical package of the social science (SPSS) version 20. Numerical variables were reported as mean± standard deviation, while categorical variables were reported as frequency (percentages). One-way ANOVA for quantitative variables and Chi square test for categorical variables were applied to compare the groups (severity of NAFLD). Pearson’s correlation was conducted to explore the correlation of uric acid with various parameters. Univariate and multivariate logistic regression was applied to explore the association between hyperuricemia and associated factor. P-value<0.05 was set as statistically significance.

## RESULTS

In this study; a total of 246 NAFLD patients were included. Out of total 87(35%) patients diagnosed to have mild stage disease, 60(24.4%) patients had moderate and 99(40.2%) patients had severe disease. Mean age of the overall participants was 53.1+/-14 years. Majority of the participants were females 128(52%). In female population most of them had severe NAFLD while majority of male patients diagnosed to have mild to moderate severity of disease. Both age and gender had shown significant association with the disease severity (P<0.05). Mean BMI was 30.61+/-5.91, compared to mild and moderate NAFLD groups; in severe NAFLD group most of the patients were overweight or obese. Mean SBP was 125.8+/-19.3 (mmHg) and mean DBP was 72.8+/-10.6 (mmHg) of NAFLD patients. Patient’s disease severity directly associated with their BMI and blood pressure (P<0.05). Only 47(19.1%) patients were smoker. Smoking habit was more observed in sever NAFLD group. Similar pattern observed for the tobacco addiction where overall 37(15%) patients were tobacco addict and majority of them found to have severe NAFLD disease. 103(41.9%) patients were physically inactive, 50(20.3%) were less active and reaming were physically active. Over 3/4^th^ of the study population had unbalanced dietary habits. All groups showed the similar pattern (P<0.05). There were 94(38.2%) patients were diabetic, 124(50.4%) patients were hypertensive, 66(26.8%) were obese, 35(14.2%) had CVD, 29(11.8%) had IHD, 13(5.3%) had CHF, 14(5.7%) had CVA and 11(4.5%) had diabetic retinopathy. Considering all the factors; the findings showed significant associations of age, gender, SBP, DBP, Tobacco addiction, diet, and obesity with the severity of NAFLD (P<0.05), Table-I.

**Table-I:** Comparison of baseline characteristics with the severity of NAFLD.

Study variables	NAFLD			Overall (n=246)	P-value
	Mild (n=87)	Moderate (n=60)	Severe (n=99)		
Age (years)	56.1±13.8	51.8±13.9	51.2±13.9	53.1±14.0	0.042
Gender					
Female	36(41.4%)	29(48.3%)	63(63.6%)	128(52%)	0.008
Male	51(58.6%)	31(51.7%)	36(36.4%)	118(48%)	
BMI (kg/m^2^)	29.2±1.8	29.5±1.6	30.1±1.9	30.61±5.91	0.002
Systolic blood pressure (mmHg)	118.9±18.3	127.8±19.5	130.7±18.4	125.8±19.3	0.0001
Diastolic blood pressure (mmHg)	70.2±10.6	72.6±9.8	75.3±10.	72.8±10.6	0.005
Smoking habit					
Yes	12(13.8%)	9(15%)	26(26.3%)	47(19.1%)	0.063
No	75(86.2%)	51(85%)	73(73.7%)	199(80.9%)	
Tobacco addiction					
Yes	7(8%)	8(13.3%)	22(22%)	37(15%)	0.024
No	80(92%)	52(86.7%)	77(77.8%)	209(85%)	
Walk 150 minutes /week					
Less	22(25.3%)	13(21.7%)	15(15.2%)	50(20.3%)	0.483
No	36(41.4%)	25(41.7%)	42(42.4%)	103(41.9%)	
Yes	29(33.3%)	22(36.7%)	42(42.4%)	93(37.8%)	
Balanced diet					
No	64(73.6%)	46(76.7%)	78(78.8%)	188(76.4%)	<0.0001
Yes	23(26.4%)	14(23.3%)	21(21.2%)	58(23.6%)	
Diabetes Mellitus					
No	64(73.6%)	38(63.3%)	50(50.5%)	152(61.8%)	0.005
Yes	23(26.4%)	22(36.7%)	49(49.5%)	94(38.2%)	
Hypertension					
No	49(56.3%)	31(51.7%)	42(42.4%)	122(49.6%)	0.399
Yes	38(43.7%)	29(48.3%)	57(57.6%)	124(50.4%)	
Obesity					
No	74(85.1%)	45(75%)	61(61.6%)	180(73.2%)	0.001
Yes	13(14.9%)	15(25%)	38(38.4%)	66(26.8%)	
CVD					
No	74(85.1%)	54(90%)	83(83.3%)	211(85.8%)	0.544
Yes	13(14.9%)	6(10%)	16(16.2%)	35(14.2%)	
IHD					
No	78(89.7%)	54(90%)	85(85.9%)	217(88.2%)	0.642
Yes	9(10.3%)	6(10%)	14(14.1%)	29(11.8%)	
CHF					
No	82(94.3%)	57(95%)	94(94.9%)	233(94.7%)	0.972
Yes	5(5.7%)	3(5%)	5(5.1%)	13(5.3%)	
CVA					
No	80(92%)	56(93.3%)	96(97%)	232(94.3%)	0.315
Yes	7(8%)	4(6.7%)	3(3%)	14(5.7%)	
PAD					
No	84(96.6%)	58(96.7%)	95(96%)	237(96.3%)	0.966
Yes	3(3.4%)	2(3.3%)	4(4%)	9(3.7%)	
Diabetic retinopathy					
No	86(98.9%)	56(93.3%)	93(93.9%)	235(95.5%)	0.173
Yes	1(1.1%)	4(6.7%)	6(6.1%)	11(4.5%)	

Data presented as mean± standard deviation or n(%); Chi square test applied; P-value<0.05 considered to be statistically significant.

Biomarkers were compared with the severity of NAFLD. Almost all the parameters have shown significant differences (P<0.001). Uric acid was relatively elevated in severe NAFLD group patients as compared to patients with mild to moderate severity of disease (7.63 vs. 6.6 vs. 5.96; P<0.001). Similarly, for HbA1c level, where mean values were (6.77 vs. 6.15 vs. 5.8; P<0.001) for severe, moderate, and mild NAFLD, respectively. Mean values of creatinine level in the groups were (0.95 vs. 0.92 vs. 0.76; P<0.001), and mean eGFR values were (58.9 vs. 59.7 vs. 59.5; P: 0.178). Mean cholesterol, triglyceride, HDL, LDL, VLDL, TB (total bilirubin), DB (direct bilirubin), GGT, SGPT, ALP, and SGOT values for the NAFLD severity groups (severe vs. moderate vs. mild) were as followed; (203.8 vs. 186.8 vs. 179.1; P<0.001), (161.2 vs. 147 vs. 118.1; P<0.001), (40.4 vs. 46.1 vs. 51.7; P<0.001), (131.9 vs. 117.7 vs. 113.2; P<0.001), (31.6 vs. 30.2 vs. 26.3; P<0.001), (1.01 vs. 0.92 vs. 0.75; P<0.001), (0.28 vs. 0.22 vs. 0.18; P<0.001), (84.4 vs. 37.3 vs. 19.5; P<0.001), (69.9 vs. 50.2 vs. 44.3; P<0.001), (121.2 vs. 95.2 vs. 80.7; P<0.001) and (55.4 vs. 41.1 vs. 37.5; P<0.001). Severe NAFLD is highly associated with elevated or abnormal biomarkers, Table-II.

**Table-II:** Relationship of biochemical parameters with the severity of NAFLD.

Parameters	NAFLD			Overall (n=246)	P-value
	Mild (n=87)	Moderate (n=60)	Severe (n=99)		
Uric Acid	5.97±1.63	6.6±1.92	7.63±1.71	6.8±1.87	<0.001
HbA1c (%)	5.8±0.78	6.15±1.37	6.77±1.01	6.29±1.11	<0.001
Creatinine	0.76±0.29	0.92±0.25	0.95±0.32	0.87±0.31	<0.001
eGFR	59.5±1.66	59.7±1.43	58.9±4.1	59.3±2.9	0.178
Cholesterol (mg/dl)	179.1±36.4	186.8±34.1	203.8±33.0	190±36.1	<0.001
Triglyceride (mg/dl)	118.1±38.3	147.01±47.4	161.2±57.7	142.5±52.4	<0.001
HDL(mg/dl)	51.7±6.91	46.1±6.01	40.4±5.68	45.8±7.90	<0.001
LDL(mg/dl)	113.2±26.4	117.7±29.4	131.9±31.4	121.8±30.3	<0.001
VLDL(mg/dl)	26.3±8.14	30.2±7.04	31.6±8.6	29.4±8.4	<0.001
TB	0.75±0.28	0.92±0.27	1.01±0.31	0.89±0.31	<0.001
DB	0.18±0.08	0.22±0.11	0.28±0.19	0.23±0.15	<0.001
GGT	19.5±6.2	37.3±4.7	84.4±26.5	49.9±33.9	<0.001
SGPT	44.3±15.2	50.2±16.6	69.9±29.3	56.0±25.0	<0.001
ALP	80.7±35.3	95.2±40.6	121.2±60.7	100.5±51.2	<0.001
SGOT	37.5±13.1	41.1±16.6	55.4±31.3	45.6±24.2	<0.001

Data presented as mean± standard deviation; ANOVA test applied; P-value<0.05 considered to be statistically significant.

**Fig.1:**
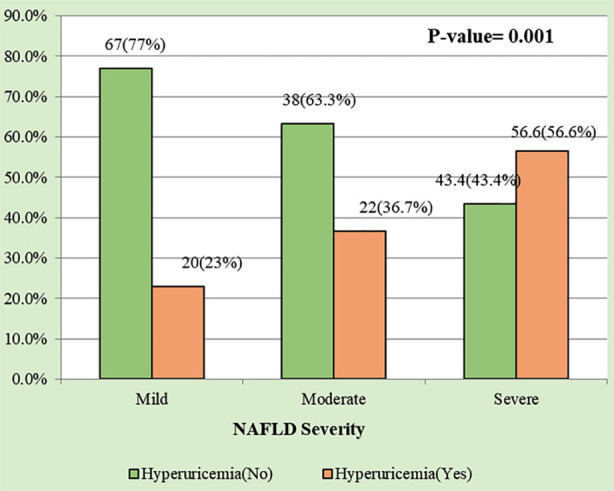
Association of Hyperuricemia with Severity of NAFLD.

The association of hyperuricemia with the severity of NAFLD was found to be significant (P: 0.001). In the mild NAFLD group, only 20 (23%) patients were diagnosed with hyperurecemia, while in the moderate group of patients, there were 22 (36.7%) patients who had hyperurecemia. The proportion is slightly higher than in the mild group, but in the severe group, there were 56 (56.6%) patients diagnosed with hyperuricemia. The pattern of findings indicated that the severity of the NAFLD disease is directly associated with elevated serum uric acid, Fig.1.

Correlations were assessed between uric acid and other study variables, including biomarkers. Correlation of uric acid with BMI (r: 0.287; P<0.001), HbA1c (r: 0.198; P: 0.002), SBP (r: 0369; P<0.001), DBP (r: 0.190; P: 0.003), Creatinine (r: 0.426; P<0.001), eGFR (r: -0.104; P: 0.104), Cholesterol (r: 0.196; P<0.002), Triglyceride (r: 0.215; P: 0.001), VLDL (r: 158; P: 0.001), HDL (r: -0.270; P< 0.001), LDL (r: 0.292; P<0.001), GGT (r: 319; P<0.001), SGOT (r: 0.149; P: 0.019), SGPT(r: 0.186; P: 0.003), DB (r: 0.221; P< 0.001), TB (r: 143; P< 0.025), and ALP (r: 0.197; P: 0.002). The majority of the parameters showed a positive correlation except age, eGFR, and HDL, as these variables have a negative relationship with uric acid, Table-III.

**Table-III:** Correlation analyses between Uric acid and various parameters

Parameters	Correlation coefficient (r)	P-value
Age	-0.007	0.911
BMI	0.287^**^	0.000
HbA1c	0.198^**^	0.002
Systolic blood pressure	0.369^**^	0.000
Diastolic blood pressure	0.190^**^	0.003
Creatinine level	0.426^**^	0.000
eGFR	-0.104	0.104
Cholesterol	0.196^**^	0.002
Triglyceride	0.215^**^	0.001
VLDL	0.158^*^	0.013
HDL	-0.270^**^	0.000
LDL	0.292^**^	0.000
GGT	0.319^**^	0.000
SGOT	0.149^*^	0.019
SGPT	0.186^**^	0.003
DB	0.221^**^	0.000
TB	0.143^*^	0.025
ALP	0.197^**^	0.002

P-value<0.05 considered to be statistically significant.

Univariate and multivariate logistic regression analysis was performed and association of hyperurecemia was assessed with the associated factors. In univariate analysis, hypertension (OR: 4.02; 95%CI: 2.32---6.95; P: 0.0001), diabetes mellitus (OR: 2.46; 95%CI: 1.45---4.18; P: 0.0001), obesity (OR: 3.87; 95%CI: 2.14---7.0; P: 0.0001), and severity of NAFLD (Severe) (OR: 4.36; 95%CI: 2.30---8.26; P: 0.0001) identified as significant factors. Similarly for the multivariate model factors like hypertension, obesity, and severity of NAFLD were found to be highly significantly associated factors, Table-IV.

**Table-IV:** Regression analysis for Hyperuricemia with associated factors.

Associated factors		Hyperuricemia		Unadjusted	Adjusted
		Yes 98(39.8%)	No 148(60.2%)	OR(95% CI); P-values	OR(95% CI); P-values
Age groups	≤50 years	43(38.7%)	68(61.3%)	Ref	
	> 50 years	55(40.7%)	80(59.3%)	1.08(0.65 ---1.81); 0.750	
Gender	Female	47(36.7%)	81(63.3%)	Ref	
	Male	51(43.2%)	67(56.8%)	0.76(0.45 ---1.27); 0.298	
Hypertension	No	29(23.8%)	93(76.2%)	Ref	Ref
	Yes	69(55.6%)	55(44.4%)	4.02(2.32 ---6.95); 0.0001	3.27(1.78 ---5.89); 0.0001
Cardiovascular Disease	No	82(38.9%)	129(61.1%)	Ref	
	Yes	16(45.7%)	19(54.3%)	1.32(0.64 ---2.27); 0.444	
Smoking	No	75(37.7%)	124(62.3%)	Ref	Ref
	Yes	23(48.9%)	24(51.1%)	1.58(0.83 ---3.00); 0.159	1.36(0.61 ---3.71); 0.447
Tobacco addiction	No	80(38.3%)	129(61.7%)	Ref	Ref
	Yes	18(48.6%)	19(51.4%)	1.53(0.75 ---3.08); 0.237	1.52(0.62 ---3.71); 0.362
Walk 150 minutes /week	No	43(41.7%)	60(58.3%)	Ref	Ref
	Yes	40(43.0%)	53(57.0%)	1.05(0.59 ---1.85); 0.858	0.88(0.46 ---1.71); 0.718
	less	15(30.0%)	35(70.0%)	0.59(0.29 ---1.23); 0.162	0.52(0.22 ---1.22); 0.132
Balanced diet	No	75(39.9%)	113(60.1%)	Ref	
	Yes	23(39.7%)	35(60.3%)	1.01(0.55 ---1.84); 0.974	
Diabetes Mellitus	No	48(31.6%)	104(68.4%)	Ref	Ref
	Yes	50(53.2%)	44(46.8%)	2.46(1.45 ---4.18); 0.001	1.49(0.81 ---2.75); 0.195
Obesity	No	56(31.1%)	124(68.9%)	Ref	Ref
	Yes	42(63.6%)	24(36.4%)	3.87(2.14 ---7.008); 0.0001	2.69(1.38 ---5.21); 0.003
Severity NAFLD	Mild	20(23.0%)	67(77.0%)	Ref	Ref
	Moderate	22(36.7%)	38(63.3%)	1.93(0.94 ---4.00); 0.073	1.71(0.78 ---3.74); 0.177
	Severe	56(56.6%)	43(43.4%)	4.36(2.20 ---8.25); 0.0001	2.95(1.43 ---6.06); 0.003

P-value for unadjusted analysis less than 0.25 is considered to be statistically significant. P-value for adjusted analysis less than 0.05 is considered to be statistically significant.

## DISCUSSION

The present study examined 246 individuals with non-alcoholic fatty liver disease (NAFLD), and the distribution of disease severity among the participants revealed a noteworthy pattern: 35% was diagnosed with mild NAFLD, 24.4% with moderate, and 40.2% with severe forms of the condition.

One of the most compelling findings of our study was the association between hyperuricemia and the severity of NAFLD. Hyperuricemia can exacerbate the progression of NAFLD from fatty liver to steatohepatitis and fibrosis through multiple mechanisms. It increases oxidative stress by releasing pro-inflammatory cytokines, which in turn leads to the injury of hepatocytes. It also regulates the activation of the NLRP3 inflammasome, which is essential for the effects of uric acid on hepatic steatosis and insulin signaling. In addition, it also leads to an increase in the production of triglycerides, which promotes hepatic lipogenesis. Hyperuricemia can accelerate NAFLD progression to NASH and fibrosis through inflammatory and oxidative stress mechanisms.^15^-^17^

The demographic profile of our study participants is consistent with existing research on NAFLD. The mean age of 53.1 years indicates that NAFLD is prevalent among middle-aged individuals, which aligns with previous research suggesting an increased risk with age.^18^,^19^ Additionally, a majority of the participants were female, comprising 52% of the study population. In contrast to our finding a systemic review and meta-analysis found NAFLD more prevalent in males.^20^ The discrepancy in gender distribution may reflect geographical differences in NAFLD prevalence or varying methodologies across studies. However, it is important to note that gender-related variations in NAFLD are still a subject of ongoing debate, with studies showing mixed results depending on the population studied.^21^

Body mass index (BMI) is a significant indicator of metabolic health and in our study the mean BMI of 30.61 among the study participants highlights the strong association between obesity and NAFLD similar to various published studies.^22^,^23^, Dietary habits emerged as another critical factor, with over three-quarters of the study population reporting unbalanced dietary patterns. Poor dietary choices, characterized by excessive consumption of unhealthy foods, have been implicated in the pathogenesis of NAFLD. Furthermore, a substantial proportion of the participants (41.9%) reported being physically inactive, reinforcing the link between sedentary lifestyles and the development of metabolic disorders such as NAFLD. Similarly, a large representative US population sample National Health and Nutrition Examination Survey (NHANE) 2017-2018 concluded that consuming healthy diet and engaging in higher levels of physical activity is linked to a decreased risk of developing NAFLD.^24^

The study also explored the comorbidities prevalent among NAFLD patients, revealing a high burden of metabolic disorders such as diabetes, hypertension, and obesity. Moreover, cardiovascular diseases (CVD), including ischemic heart disease (IHD), congestive heart failure (CHF), and stroke, were also observed in a notable proportion of our study participants aligned to previous studies.^25^,^26^

The presence of these comorbidities significantly increases the risk of more severe forms of NAFLD and its complications, including liver fibrosis, cirrhosis, and even hepatocellular carcinoma. Additionally, cardiovascular diseases (CVD), including ischemic heart disease (IHD), congestive heart failure (CHF), and stroke, observed in a notable proportion of participants in our study, supporting findings from another study suggests a bidirectional relationship between NAFLD and cardiovascular disease.^27^

Diabetic retinopathy (DR), a complication of diabetes, was present in a smaller yet significant subset of patients. DR is associated with NAFLD is still matter of debate and results are inconclusive, however one study established association in univariate analysis but not in multivariate analysis.^28^

Moreover, the distribution of disease severity among the participants revealed a noteworthy pattern: 35% were diagnosed with mild NAFLD, 24.4% with moderate, and 40.2% with severe forms of the condition. Importantly, the analysis identified that age, gender, blood pressure, tobacco addiction, dietary habits, and obesity were significantly associated with disease progression. These findings reinforce the multifactorial nature of NAFLD, where genetic, lifestyle, and environmental factors all contribute to the severity of the disease. Notably, the significant association of hypertension and obesity with severe NAFLD observed in our study corroborates findings from other recent studies, also emphasize the need for managing these risk factors to prevent or slow down disease progression.^29^,^30^

### Limitations:

While the study sheds light on the frequency, associations between various factors and the severity of non-alcoholic fatty liver disease (NAFLD), its limitations warrant consideration. With a sample size of 246 patients, the study’s generalizability may be limited, and selection bias could arise from recruitment within a specific healthcare setting. Furthermore, it was retrospectively designed study and cross-sectional nature limits causal inference.

## CONCLUSION

A notable prevalence of hyperuricemia was observed among NAFLD patients, particularly evident in middle-aged individuals and females. Therefore, incorporating uric acid evaluations into routine clinical practice may offer an opportunity to improve NAFLD management and alleviate metabolic complications related with hyperuricemia.

### Recommendation:

From a clinical perspective, it is recommended to regularly monitor serum uric acid levels in NAFLD patients, as it is a modifiable risk factor that can that can aid in the management of NAFLD. Furthermore, given its association with metabolic syndrome and cardiovascular diseases, assessing uric acid can help in the early detection of conditions like diabetes, hypertension, and dyslipidemia. Additionally, this assessment can guide dietary interventions, such as a low purine diet, and inform the need for pharmacological treatment.

### Authors’ Contribution:

**ALH:** Designed, did data collection, statistical analysis, and manuscript writing, and is responsible for the integrity of research and final approval of manuscript.

**SA:** Did conception and design, manuscript writing, and revised it critically for important intellectual content.

**AH** and **ZS:** Did data collection, manuscript writing, and review of the article.

All authors have approved the final version.
